# GWAS-Informed Candidate Genetic Variants and Gene–Gene/Gene–Environment Interaction Patterns Associated with Intervertebral Disc Degeneration: A Chinese Han Case–Control Study

**DOI:** 10.3390/genes17080967

**Published:** 2026-08-18

**Authors:** Ridan Lei, Mengyuan Zhang, Manjun Luo, Xiaorui Ruan, Jianhui Wei, Ziye Li, Jiabi Qin

**Affiliations:** 1 Department of Epidemiology and Health Statistics, Xiangya School of Public Health, Central South University, Changsha 410013, China; 2 Department of Epidemiology and Health Statistics, School of Public Health, Kunming Medical University, Kunming 650500, China

**Keywords:** intervertebral disc degeneration, single-nucleotide polymorphism, genetic susceptibility, case–control study, Chinese Han population, haplotype, gene–gene interaction, gene–environment interaction

## Abstract

Background/Objectives: Intervertebral disc degeneration (IDD) is a multifactorial spinal degenerative condition influenced by inherited susceptibility and environmental exposures. Genetic evidence from Chinese Han populations remains limited. This study aimed to evaluate GWAS-informed and previously reported candidate single-nucleotide polymorphisms (SNPs) associated with IDD and to explore potential gene–gene and gene–environment interaction patterns. Methods: A hospital-based case–control study was conducted in 1951 unrelated Chinese Han participants, including 929 patients with magnetic resonance imaging-confirmed IDD and 1022 control subjects without evident IDD. Candidate SNPs were genotyped using the MassARRAY platform. Multivariable logistic regression was used to assess genetic associations under different genetic models, and linkage disequilibrium, haplotype associations, additive and multiplicative interactions, and generalized multifactor dimensionality reduction (GMDR) were further evaluated. Results: Of the 60 initially selected candidate SNPs, 49 passed quality control and were retained for association analyses. Several variants showed nominal associations with IDD susceptibility. Representative associations included *MMP3* rs591058 under the recessive model (OR = 1.37, 95% CI: 1.06–1.79, *p* = 0.019) and *VDR* rs2228570 under the dominant model (OR = 1.52, 95% CI: 1.10–2.11, *p* = 0.012), whereas the *CCDC26* rs6651255–rs7816342 AC haplotype showed a protective association (OR = 0.87, 95% CI: 0.76–0.99, *p* = 0.046). Exploratory interaction analyses suggested possible gene–gene interactions involving *ACAN*, *IL1B*, *VDR*, *CASP3*, and *CEP162/RIPPLY2*, as well as gene–environment interactions involving smoking, weight-bearing workload, and duration of bending work. Conclusions: This study provides preliminary genetic epidemiological evidence that GWAS-informed candidate variants and exploratory interaction patterns may be associated with IDD susceptibility in the Chinese Han population. These findings require further replication and functional validation.

## 1. Introduction

Intervertebral disc degeneration (IDD) is a common degenerative condition of the spine and is closely related to low-back pain, intervertebral disc herniation, spinal stenosis, and other spinal degenerative disorders [1,2]. The intervertebral disc is composed mainly of the nucleus pulposus, annulus fibrosus, and cartilage endplates, which together maintain spinal stability and biomechanical function [3,4]. Degenerative changes in these structures may lead to extracellular matrix degradation, impaired disc hydration, inflammation, cell senescence, apoptosis, and structural failure of the disc [5,6]. Because spine-related pain and degenerative spinal disorders contribute substantially to disability and health-care burden worldwide, identifying susceptibility factors for IDD has important public health and clinical relevance [7].

IDD is generally considered a multifactorial disease. Ageing, biomechanical loading, metabolic dysfunction, occupational workload, and other environmental or lifestyle factors may contribute to IDD-related phenotypes [8,9,10]. Smoking has also been specifically implicated in intervertebral disc degeneration [11]. However, environmental exposures alone cannot fully explain interindividual differences in IDD susceptibility. Family-based and heritability studies have suggested a substantial inherited component of lumbar disc degeneration and related spinal phenotypes [12,13,14], while magnetic resonance imaging studies of identical twins provide additional evidence for genetic susceptibility [15].

Previous candidate gene studies have reported associations between IDD and structural, degradative, and inflammatory genes [16,17,18]. Reviews have summarized a broader genetic architecture involving extracellular matrix regulation, inflammation, apoptosis, oxidative stress, vitamin D signalling, bone remodelling, and skeletal development [19,20]. Variants in *VDR* and *GDF5* represent two frequently investigated examples of candidate genetic factors related to disc degeneration [21,22]. Collectively, these findings suggest that IDD is a biologically heterogeneous disorder involving multiple interacting molecular pathways rather than a single pathogenic mechanism.

In recent years, genome-wide association studies (GWASs) and large-scale biobank analyses have provided a more systematic framework for identifying genetic loci associated with IDD-related spinal phenotypes [23,24,25]. Specifically, a genome-wide meta-analysis identified a locus associated with Modic changes [23], while a sequence variant at the *CCDC26/GSDMC* locus was associated with sciatica caused by lumbar disc herniation [26]. Variants in *SLC13A1* and the sulphate metabolism-related *CHST3* pathway were subsequently associated with intervertebral disc disorders [27]. Large-scale meta-analyses have also identified loci associated with chronic back pain [28] and substantially expanded the genetic landscape of lumbar disc herniation using data from three large biobanks [29]. Although some studies have included non-European populations, much of the available large-scale GWAS evidence has been generated from populations of European ancestry. The transferability of these findings to Chinese Han populations cannot be assumed because allele frequencies, linkage disequilibrium structures, genetic backgrounds, and environmental or occupational exposures may differ across populations [19,20]. Independent validation is therefore necessary to determine whether previously reported susceptibility loci are also relevant to IDD in Chinese Han individuals.

Despite these advances, previous genetic studies of IDD and related spinal phenotypes have several limitations [19,20]. First, the definitions of IDD and related outcomes have varied across studies, with some studies using imaging-based disc degeneration and others using clinical phenotypes such as low-back pain, sciatica, or lumbar disc herniation, thereby increasing phenotypic heterogeneity. Second, many candidate gene studies included relatively small samples and evaluated only a limited number of variants, which may have reduced statistical power and contributed to inconsistent findings. Third, the replication of previously reported loci across different ancestral populations remains insufficient. Finally, most previous studies focused primarily on the main effects of individual variants, whereas the combined contribution of multiple susceptibility loci and environmental exposures has been less extensively investigated.

We therefore adopted an integrated GWAS-informed and candidate gene strategy [19,20,29]. GWAS-derived loci provide relatively hypothesis-agnostic evidence of population-level genetic susceptibility and may identify previously unrecognized genomic regions. In contrast, previously reported candidate variants provide pathway-based evidence concerning genes with established or biologically plausible roles in disc degeneration, including extracellular matrix metabolism, inflammation, apoptosis, vitamin D signalling, bone remodelling, skeletal development, and circadian regulation. These two sources of evidence are complementary rather than mutually exclusive. Combining them enabled us to evaluate both large-scale genetic signals and biologically plausible candidate variants and to assess their relevance in a Chinese Han population.

In addition to the main effects of individual variants, IDD susceptibility may be influenced by interactions among multiple genetic loci and between genetic variants and environmental exposures. Previous studies have suggested that *MMP3* and *VDR* polymorphisms may modify the effects of occupational loading and other mechanical exposures on lumbar disc degeneration, while *MMP3* rs591058 may interact with occupational risk factors in lumbar disc herniation [30,31]. Smoking may impair disc nutrition and increase inflammatory or oxidative stress, whereas prolonged bending and heavy weight-bearing work may aggravate mechanical damage to the intervertebral disc [10,11]. Nevertheless, previous interaction studies have generally evaluated only a limited number of genes and environmental factors, and evidence regarding multilocus and higher-order interaction patterns remains insufficient, particularly in Chinese populations.

Accordingly, the present study was designed as a candidate locus validation and exploratory interaction study rather than a de novo genome-wide discovery study. We evaluated GWAS-informed loci and previously reported IDD-associated candidate variants in a Chinese Han case–control population. We further assessed linkage disequilibrium and haplotype associations and explored potential gene–gene and gene–environment interaction patterns related to IDD susceptibility.

## 2. Materials and Methods

### 2.1. Ethics Statement

This study was conducted in accordance with the Declaration of Helsinki and was approved by the Ethics Committee of the Affiliated Nanhua Hospital, University of South China (protocol code: 2023-ky-003; date of approval: 1 December 2023). All participants were informed of the purpose, procedures, potential risks, and benefits of the study before enrollment. Written informed consent was obtained from all participants.

### 2.2. Study Design and Participants

A hospital-based case–control study was conducted among unrelated Chinese Han individuals. In total, 1951 participants were included in the final analysis, comprising 929 patients with IDD and 1022 control subjects.

Patients with IDD were recruited from the Department of Spine Surgery, Affiliated Nanhua Hospital, University of South China, between December 2023 and June 2025. IDD was diagnosed based on clinical assessment and lumbar magnetic resonance imaging (MRI). Lumbar MRI scans were evaluated at five intervertebral disc levels, from L1/L2 to L5/S1, using sagittal T2-weighted images. Each intervertebral disc level was graded separately according to the Pfirrmann grading system. For participant-level classification, the highest Pfirrmann grade observed across the five assessed lumbar levels was used to represent the participant’s overall degeneration status. Participants were classified as IDD cases if at least one assessed lumbar intervertebral disc had a Pfirrmann grade of III–V.

Control subjects were recruited during the same period from individuals who underwent lumbar MRI for routine health examinations or non-spinal conditions. Controls were required to have Pfirrmann grades I–II at all five assessed lumbar intervertebral disc levels and no evident lumbar disc herniation or other severe spinal degenerative disease. Individuals with any assessed lumbar intervertebral disc graded III or higher were not included in the control group.

All participants were Chinese Han individuals and were able to complete the questionnaire survey, MRI examination, and peripheral blood collection. Individuals were excluded if they had a history of major spinal trauma, spinal infection, spinal surgery, spinal tumour, obvious spinal deformity, severe lower-limb joint disease affecting spinal mechanical loading, malignant tumour, connective tissue disease, known genetic syndrome, chromosomal abnormality, or other conditions considered unsuitable for inclusion by the investigators.

### 2.3. Questionnaire Survey and Environmental Exposure Assessment

Demographic characteristics and environmental exposure information were collected using a structured face-to-face questionnaire administered by trained investigators. The collected variables included age, sex, ethnicity, education level, marital status, smoking status, alcohol drinking, physical activity, dietary habits, occupational type, occupational workload, duration of bending work, history of disease, and family history of disc-related disease.

Smoking exposure was categorized according to the average number of cigarettes smoked per day multiplied by the number of years of smoking. Weight-bearing workload and duration of bending work were classified based on occupational exposure information collected through the questionnaire. Family history was defined as a self-reported history of disc-related disease in first-degree relatives. These environmental variables were used in the subsequent multivariable and gene–environment interaction analyses.

### 2.4. Candidate SNP Selection

Candidate SNPs were selected using an integrated, multistep strategy based on the present GWAS meta-analysis and evidence from a systematic review. First, publicly available GWAS summary statistics from FinnGen, the UK Biobank, and GCST90436667 were harmonized and meta-analyzed [32,33]. SNPs with a meta-analysis *p*-value < 5 × 10^−6^, concordant effect directions across the three datasets, and acceptable heterogeneity (I^2^ < 70%) were considered suggestively associated variants. Meta-analysis was performed using standard inverse variance-weighted methods implemented in METAL [34]. These variants were subsequently prioritized according to positional mapping, expression quantitative trait locus mapping, chromatin interaction mapping, and biological relevance using FUMA and related annotation resources [35].

Second, evidence from previous candidate gene studies and GWASs of IDD and related spinal phenotypes was systematically reviewed [19,20]. Variants supported only by small or low-quality studies or by studies with unclear phenotype definitions were excluded. Variants with a minor allele frequency < 0.05 in East Asian populations were excluded, and one representative SNP was preferentially retained from sets of highly correlated variants within the same linkage disequilibrium block (r^2^ > 0.8). Biological relevance to extracellular matrix homeostasis, matrix degradation and remodelling, inflammation, apoptosis, oxidative stress, vitamin D signalling, bone remodelling, skeletal development, metabolic regulation, and circadian regulation was also considered. Technical feasibility for multiplex genotyping using the MassARRAY platform was evaluated before final inclusion.

After merging and deduplicating the two evidence sources, 60 candidate SNPs representing 46 candidate genes or loci were selected for genotyping. After genotyping quality control and Hardy–Weinberg equilibrium evaluation in controls, 49 SNPs were retained for the main association analyses. The detailed selection rationale for each initially selected SNP, including the associated or nearest gene or locus, biological category, evidence type, source phenotype or study, supporting citation, East Asian allele frequency, and post-genotyping quality-control status, is provided in Supplementary Table S7.

### 2.5. DNA Extraction and Genotyping

Approximately 5 mL of peripheral venous blood was collected from each participant into an EDTA anticoagulant tube. Blood samples were transported to the laboratory under low-temperature conditions, centrifuged, aliquoted, and stored at −80 °C until DNA extraction and genotyping.

Genomic DNA was extracted from peripheral blood cell samples using standard procedures. SNP genotyping was performed using the Agena MassARRAY time-of-flight mass spectrometry platform (Agena Bioscience, San Diego, CA, USA). Briefly, multiplex PCR primers and single-base extension primers were designed for the target SNP regions. PCR amplification, shrimp alkaline phosphatase treatment, single-base extension reactions, resin purification, and SpectroCHIP spotting were performed according to the manufacturer’s protocol. The purified products were analyzed using the MassARRAY Analyzer 4.0 system, and genotype calls were generated using MassARRAY TYPER 4.0 software.

SNPs that failed genotyping quality control or showed significant deviation from Hardy–Weinberg equilibrium in controls after false discovery rate correction were excluded from the main association analyses.

### 2.6. Statistical Analysis

Questionnaire data were entered using EpiData 3.0 (EpiData Association, Odense, Denmark) with logical verification rules. Double data entry was independently performed by two trained investigators, and discrepancies were checked against the original questionnaires. Missing or questionable records were corrected by reviewing the original questionnaire or by telephone follow-up when necessary. For a small number of randomly missing environmental variables or covariates, multivariable predictive imputation was performed using the missForest package (version 1.4) in R software.

Statistical analyses were performed using IBM SPSS Statistics version 26.0, R version 4.0.3, Haploview version 4.2, and GMDR version 1.0. Continuous variables were expressed as the mean ± standard deviation or median with interquartile range, as appropriate. Categorical variables were expressed as numbers and percentages. Differences between cases and controls were evaluated using the independent-samples *t*-test, Mann–Whitney U test, chi-square test, or Fisher’s exact test, as appropriate.

Hardy–Weinberg equilibrium was assessed among controls using the chi-square test. To account for the evaluation of multiple loci, Benjamini–Hochberg false discovery rate-adjusted Q values were also calculated. SNPs showing significant deviation from Hardy–Weinberg equilibrium after false discovery rate correction or failing genotyping quality control were excluded from the main association analyses. Associations between SNPs and IDD risk were evaluated using logistic regression models under codominant, dominant, recessive, and additive genetic models. Odds ratios (ORs) and 95% confidence intervals (CIs) were calculated. Multivariable models were adjusted for potential confounders, including age, body mass index, education level, smoking exposure, family history of disc-related disease, weight-bearing workload, and duration of bending work, unless otherwise specified.

Linkage disequilibrium between SNPs was assessed using Haploview 4.2. Haplotype frequencies were estimated, and haplotype associations with IDD risk were evaluated using logistic regression models.

Gene–gene and gene–environment interactions were evaluated on both additive and multiplicative scales. Additive interaction was assessed using the relative excess risk due to interaction (RERI), attributable proportion due to interaction (AP), and synergy index (S) [36,37]. Multiplicative interaction was assessed by including product terms in logistic regression models. GMDR was further used to explore potential higher-order interaction models [38]. Cross-validation consistency, testing balanced accuracy, and sign testing were used to evaluate model stability.

Because this study was designed as a candidate locus validation and exploratory interaction study, two-sided *p*-values < 0.05 were considered nominally significant for the association and interaction analyses. Given the number of candidate variants, genetic models, SNP–SNP combinations, and SNP–environment combinations evaluated, these results were not interpreted as confirmatory evidence. The gene–gene and gene–environment interaction analyses were considered exploratory and hypothesis-generating. Estimates based on rare genotypes, sparse genotype combinations, or wide confidence intervals were interpreted cautiously. Detailed association and interaction results are provided in the Supplementary Materials.

## 3. Results

### 3.1. Characteristics of the Study Population

A total of 1951 unrelated Chinese Han participants were included in the final analysis, including 929 patients with MRI-confirmed IDD and 1022 control subjects. All participants completed the questionnaire survey, peripheral venous blood collection, and candidate SNP genotyping.

The baseline characteristics of the study population are shown in Table 1. Sex and education level were comparable between IDD cases and controls. Age, BMI, family history of disc-related disease, smoking exposure, weight-bearing workload, and duration of bending work differed between the two groups. These variables were therefore considered as potential confounders or exposure factors and were adjusted for in subsequent multivariable or interaction analyses when appropriate.

### 3.2. Genotyping Quality Control

A total of 60 candidate SNPs were initially selected from GWAS-informed and previously reported IDD-associated loci. After quality control and Hardy–Weinberg equilibrium evaluation, 49 SNPs were retained for association analyses. The excluded SNPs were removed because of genotyping failure, low genotyping quality, or deviation from Hardy–Weinberg equilibrium in controls. Detailed genotyping quality control, Hardy–Weinberg equilibrium, and inclusion status are provided in Supplementary Table S2.

### 3.3. Association Between Candidate SNPs and IDD Risk

The main nominal associations between candidate SNPs and IDD susceptibility are summarized in Table 2. The complete results under different genetic models are provided in Supplementary Table S1.

After adjustment for potential confounders, several candidate variants showed nominal associations with IDD susceptibility. Among the risk-associated variants, *ACAN* rs698621 was associated with increased IDD risk in the codominant comparison (TG vs. TT: OR = 1.43, 95% CI: 1.01–2.02, *p* = 0.043). *MMP3* rs591058 was associated with increased IDD risk under both the recessive model (OR = 1.37, 95% CI: 1.06–1.79, *p* = 0.019) and the additive model (OR = 1.24, 95% CI: 1.02–1.51, *p* = 0.032). *IL1B* rs16944 was associated with increased IDD risk under the recessive model (OR = 1.36, 95% CI: 1.03–1.81, *p* = 0.033). *CASP3* rs4647693 showed an association with increased IDD risk under the dominant model (OR = 4.76, 95% CI: 1.51–15.07, *p* = 0.008), although the wide confidence interval suggests limited precision. *VDR* rs2228570 was associated with increased IDD risk under the dominant model (OR = 1.52, 95% CI: 1.10–2.11, *p* = 0.012) and the additive model (OR = 1.22, 95% CI: 1.00–1.48, *p* = 0.047). *ARNTL* (*BMAL1*) rs7949336 was also associated with increased IDD risk under the dominant model (OR = 1.49, 95% CI: 1.06–2.09, *p* = 0.021).

In contrast, several variants showed nominal protective associations with IDD. *CCDC26* rs6651255 was associated with reduced IDD risk under the dominant model (OR = 0.51, 95% CI: 0.29–0.91, *p* = 0.021) and the additive model (OR = 0.76, 95% CI: 0.59–0.98, *p* = 0.034). *CCDC26* rs7816342 was associated with reduced IDD risk under the dominant model (OR = 0.48, 95% CI: 0.28–0.84, *p* = 0.009). *CEP162/RIPPLY2* rs62420748 showed a protective association under the recessive model (OR = 0.58, 95% CI: 0.35–0.96, *p* = 0.034) and the additive model (OR = 0.77, 95% CI: 0.61–0.96, *p* = 0.024). *LRP5* rs3781579 was associated with reduced IDD risk under the dominant model (OR = 0.62, 95% CI: 0.40–0.96, *p* = 0.032) and the additive model (OR = 0.64, 95% CI: 0.42–0.98, *p* = 0.039). *GDF5* rs143383 was associated with lower IDD risk in the AA versus GG comparison (OR = 0.56, 95% CI: 0.33–0.95, *p* = 0.032) and under the additive model (OR = 0.76, 95% CI: 0.61–0.96, *p* = 0.018).

### 3.4. Linkage Disequilibrium and Haplotype Analysis

Linkage disequilibrium analysis showed that several SNP pairs were in strong linkage disequilibrium within the same gene regions. Strong linkage disequilibrium was observed for *ACAN* rs1042630–rs3825994, *CHST3* rs1871452–rs4148946, and *CCDC26* rs6651255–rs7816342, with r^2^ values ≥ 0.80.

Haplotype analysis further suggested that the *CCDC26* rs6651255–rs7816342 haplotype was associated with IDD susceptibility. The AC haplotype formed by *CCDC26* rs6651255–rs7816342 was associated with a reduced risk of IDD (OR = 0.87, 95% CI: 0.76–0.99, *p* = 0.046). No statistically significant associations were observed for the other haplotypes. The main linkage disequilibrium and haplotype results are summarized in Table 3, and detailed results are provided in Supplementary Table S3.

### 3.5. Gene–Gene Interaction Analysis

Exploratory gene–gene interaction analyses were performed for selected candidate variants that showed nominal or suggestive associations with IDD in the main-effect analyses. After adjustment for age, BMI, education level, smoking exposure, family history of disc-related disease, weight-bearing workload, and duration of bending work, several SNP combinations showed potential interaction signals.

On the additive scale, *ACAN* rs698621 showed positive interactions with *CASP3* rs4647693, *VDR* rs2228570, and *CEP162/RIPPLY2* rs62420748, with RERI values of 1.29 (95% CI: 0.22–2.36), 0.74 (95% CI: 0.26–1.22), and 0.49 (95% CI: 0.08–0.91), respectively. *IL1B* rs16944-*VDR* rs2228570 and *CASP3* rs4647693-*CEP162/RIPPLY2* rs62420748 also showed positive additive interaction signals, with RERI values of 0.90 (95% CI: 0.69–1.11) and 0.85 (95% CI: 0.56–1.14), respectively.

On the multiplicative scale, *ACAN* rs698621–*CEP162/RIPPLY2* rs62420748 (interaction OR = 2.08, 95% CI: 1.07–4.02, *p* = 0.030), *IL1B* rs16944–*VDR* rs2228570 (interaction OR = 5.55, 95% CI: 2.34–13.15, *p* < 0.001), and *VDR* rs2228570–*GDF5* rs143383 (interaction OR = 3.96, 95% CI: 1.05–14.93, *p* = 0.042) suggested synergistic multiplicative interactions. In contrast, *MMP3* rs591058–*CEP162/RIPPLY2* rs62420748 (interaction OR = 0.18, 95% CI: 0.07–0.46, *p* < 0.001), *CASP3* rs4647693–*VDR* rs2228570 (interaction OR = 0.05, 95% CI: 0.0001–0.55, *p* = 0.015), and *LRP5* rs3781579–*CEP162/RIPPLY2* rs62420748 (interaction OR = 0.35, 95% CI: 0.15–0.84, *p* = 0.019) suggested antagonistic multiplicative interactions. Interaction estimates based on sparse genotype combinations or wide confidence intervals should be interpreted cautiously. The key gene–gene interaction results are summarized in Table 4, and detailed results are provided in Supplementary Table S4.

### 3.6. Gene–Environment Interaction Analysis

Gene–environment interaction analyses were performed to evaluate whether selected environmental exposures modified the associations between candidate variants and IDD risk. After adjustment for age, BMI, and education level, several SNP–environment combinations showed exploratory interaction signals.

On the additive scale, *MMP3* rs591058–smoking (RERI = 0.85, 95% CI: 0.15–1.56), *IL1B* rs16944–duration of bending work (RERI = 1.33, 95% CI: 0.04–2.61), *CASP3* rs4647693–smoking (RERI = 2.46, 95% CI: 0.37–4.55), and *ARNTL* (*BMAL1*) rs7949336–duration of bending work (RERI = 1.42, 95% CI: 0.14–2.70) suggested potential synergistic additive interactions.

On the multiplicative scale, *MMP3* rs591058–smoking showed a synergistic interaction with IDD risk (interaction OR = 1.94, 95% CI: 1.01–3.73, *p* = 0.045). *CASP3* rs4647693–weight-bearing workload also showed a synergistic multiplicative interaction (interaction OR = 9.51, 95% CI: 1.90–47.53, *p* = 0.006). In contrast, *LRP5* rs3781579–weight-bearing workload (interaction OR = 0.38, 95% CI: 0.16–0.90, *p* = 0.027) and *GDF5* rs143383–smoking (interaction OR = 0.37, 95% CI: 0.17–0.81, *p* = 0.013) suggested antagonistic multiplicative interactions. The key gene–environment interaction results are summarized in Table 5, and detailed results for the selected interaction signals are provided in Supplementary Table S5.

### 3.7. GMDR Analysis

GMDR analysis was further performed to explore high-order interaction patterns. For gene–gene interactions, the model including *ACAN* rs698621, *IL1B* rs16944, *VDR* rs2228570, and *CEP162/RIPPLY2* rs62420748 showed relatively favourable model performance and cross-validation consistency among the candidate models. For gene–environment interactions, models involving *ARNTL* (*BMAL1*) rs7949336, smoking, weight-bearing workload, and duration of bending work showed relatively stable performance. Detailed GMDR model performance, including training balanced accuracy, testing balanced accuracy, cross-validation consistency, and sign-test *p*-values, is provided in Supplementary Table S6.

## 4. Discussion

In this Chinese Han case–control study, we evaluated the associations of GWAS-informed and previously reported candidate genetic variants with IDD susceptibility. Several variants in genes related to extracellular matrix metabolism, inflammation, apoptosis, vitamin D signalling, bone remodelling, and circadian rhythm regulation showed nominal associations with IDD risk. We also observed a protective haplotype in the *CCDC26* region and identified several exploratory gene–gene and gene–environment interaction patterns. These findings suggest that IDD susceptibility may be influenced not only by individual genetic variants but also by their combined effects with occupational and lifestyle exposures.

The associations observed for *ACAN* and *MMP3* support the potential importance of extracellular matrix homeostasis in IDD. *ACAN* encodes aggrecan, a major proteoglycan component of the intervertebral disc extracellular matrix that contributes to disc hydration and resistance to compressive loading [39,40,41]. *MMP3* encodes matrix metalloproteinase-3, which participates in extracellular matrix degradation and remodelling [42,43]. The observed associations involving *ACAN* rs698621 and *MMP3* rs591058 are therefore biologically plausible, although statistical association alone does not establish direct causality.

Inflammatory and vitamin D-related pathways may also contribute to IDD susceptibility. *IL1B* is a key inflammatory cytokine gene, and IL-1β-related signalling may promote extracellular matrix catabolism, inflammatory responses, cellular senescence, and pain-related degenerative changes in the intervertebral disc [44,45]. *VDR* is involved in bone metabolism, cartilage homeostasis, and musculoskeletal function [46,47]. *VDR* rs2228570, also known as the FokI polymorphism, affects the translation initiation region of the *VDR* gene. These findings strengthen the biological plausibility of the associations observed for *IL1B* and *VDR*.

*GDF5* is involved in skeletal and cartilage development and has been implicated in the regulation of intervertebral disc structure and homeostasis [48,49,50]. The location of rs143383 in the 5′ untranslated regulatory region and its previously reported associations with lumbar disc degeneration provide biological support for the present finding. Single-cell analysis has also implicated Wnt/Ca^2+^ signalling, inflammation, and apoptosis in nucleus pulposus degeneration [51], providing pathway-level support for the potential involvement of *LRP5* in IDD. Nevertheless, direct variant-specific functional evidence remains limited.

*CASP3* encodes caspase-3, an important executor of apoptosis, and caspase-family-mediated cell death contributes to nucleus pulposus and annulus fibrosus degeneration [52,53]. The relatively strong association between *CASP3* rs4647693 and IDD risk should be interpreted cautiously because of its wide confidence interval. *ARNTL* (*BMAL1*) is a core circadian-clock gene, and experimental evidence suggests that BMAL1-related signalling may regulate nucleus pulposus cell apoptosis and disc homeostasis [54]. These pathway-level findings support the biological relevance of *CASP3* and *ARNTL/BMAL1* but do not demonstrate that the specific variants identified in the present study are directly causal.

The observed gene–environment interaction patterns are also biologically plausible. Smoking may impair disc nutrition and promote inflammatory or oxidative stress [10,11], whereas cumulative physical workload, repeated weight-bearing, and prolonged bending may aggravate mechanical damage to the lumbar spine [55].

The gene–gene and gene–environment interaction analyses provide preliminary evidence that IDD susceptibility may not be fully explained by the main effects of individual variants. Additive and multiplicative interaction measures and GMDR provide complementary approaches for assessing potential interactions between genetic and environmental factors [36,37,38]. Nevertheless, because multiple genetic models, SNP–SNP combinations, and SNP–environment combinations were examined, the possibility of false-positive findings cannot be excluded. These interaction results should therefore be regarded as exploratory and hypothesis-generating rather than confirmatory. Replication in independent cohorts with prespecified interaction models, adequate sample sizes within each genotype–exposure stratum, and appropriate control of multiplicity is required before these interaction signals can be considered robust.

This study has several strengths. First, the candidate SNPs were selected based on GWAS-informed loci and previously reported IDD-associated variants, which increased the biological plausibility of the genetic markers evaluated. Second, this study included a relatively large Chinese Han case–control sample with MRI-based IDD classification. Third, both main genetic effects and interaction patterns were evaluated, allowing for a more comprehensive assessment of genetic susceptibility and environmental modification. Fourth, the use of haplotype analysis and GMDR provided complementary perspectives on multi-locus and multi-factor patterns.

Several limitations should be acknowledged. First, this was a hospital-based case–control study. Patients recruited from a spine-surgery department may have had more severe symptoms or degeneration than individuals with IDD in the general population, and selection bias cannot be fully excluded. Cases and controls also differed in age and several baseline characteristics. Although multivariable adjustment was performed, residual confounding due to unmeasured or imprecisely measured factors may remain. In addition, environmental and occupational exposures were collected mainly through self-reported questionnaires and may therefore have been affected by recall bias, measurement error, or exposure misclassification.

Second, several main-effect and interaction estimates had relatively wide confidence intervals. Although the overall sample size was relatively large for a candidate locus case–control study, the effective sample size was substantially smaller for rare genotype groups and for specific genotype–genotype or genotype–exposure combinations. Low minor-allele frequencies, uncommon homozygous genotypes, and sparse interaction strata may therefore have reduced statistical precision and contributed to unstable or inflated effect estimates. Large ORs accompanied by very wide confidence intervals should consequently be interpreted as preliminary signals rather than precise estimates of effect magnitude.

Third, multiple candidate SNPs, genetic models, and interaction combinations were evaluated. Although the analyses were explicitly treated as exploratory, false positive findings cannot be excluded. The generalizability of the results should also be considered. The study participants were Chinese Han individuals recruited from a single hospital, and the observed associations may not be directly transferable to populations with different allele frequencies, linkage disequilibrium structures, genetic backgrounds, environmental exposures, occupational patterns, or clinical characteristics. Finally, the findings were not validated in an independent external cohort. Replication in independent Chinese cohorts and other ethnic populations, together with variant-specific functional studies, is required.

## 5. Conclusions

In conclusion, this Chinese Han case–control study identified several nominal associations between GWAS-informed or previously reported candidate variants and IDD susceptibility. A protective *CCDC26* haplotype and several potential gene–gene and gene–environment interaction patterns were also observed. These findings provide preliminary and hypothesis-generating genetic epidemiological evidence concerning extracellular matrix regulation, inflammation, apoptosis, vitamin D signalling, bone remodelling, circadian regulation, and environmental modification in IDD. However, the observed associations should not be interpreted as confirmatory or causal. Independent replication in larger and more diverse populations and variant-specific functional studies are required.

## Figures and Tables

**Table 1:** Baseline characteristics of study population.

Characteristics	Controls (*n* = 1022)	IDD Cases (*n* = 929)	*p*-Value
Sex			0.914
Male	530 (51.9%)	485 (52.2%)	
Female	492 (48.1%)	444 (47.8%)	
Age, years			0.047
<50	520 (50.9%)	430 (46.3%)	
≥50	502 (49.1%)	499 (53.7%)	
BMI, kg/m^2^			0.024
<18.5	35 (3.4%)	25 (2.7%)	
18.5–<24.0	690 (67.5%)	575 (61.9%)	
24.0–<28.0	260 (25.4%)	290 (31.2%)	
≥28	37 (3.6%)	39 (4.2%)	
Education level			0.842
Primary school or below	230 (22.5%)	215 (23.1%)	
Middle school	290 (28.4%)	270 (29.1%)	
College or above	502 (49.1%)	444 (47.8%)	
Family history of IDD			0.007
No	812 (79.5%)	689 (74.2%)	
Yes	210 (20.5%)	240 (25.8%)	
Smoking exposure			0.040
Never	650 (63.6%)	540 (58.1%)	
1–100	230 (22.5%)	220 (23.7%)	
101–200	100 (9.8%)	120 (12.9%)	
>200	42 (4.1%)	49 (5.3%)	
Weight-bearing workload			<0.001
Light	720 (70.5%)	550 (59.2%)	
Moderate	250 (24.5%)	290 (31.2%)	
Heavy	52 (5.1%)	89 (9.6%)	
Duration of bending work			<0.001
<2 h	550 (53.8%)	420 (45.2%)	
2–4 h	300 (29.4%)	320 (34.4%)	
≥4 h	172 (16.8%)	189 (20.3%)	

Note: Data are presented as *n* (%). Differences between cases and controls were assessed using the chi-square test. IDD, intervertebral disc degeneration; BMI, body mass index.

**Table 2:** Main nominal associations between candidate SNPs and IDD risk in Chinese Han population.

Gene	SNP	Genetic Model	OR	95% CI	*p*-Value	Direction
*ACAN*	rs698621	TG vs. TT	1.43	1.01–2.02	0.043	Increased risk
*MMP3*	rs591058	Recessive model	1.37	1.06–1.79	0.019	Increased risk
*MMP3*	rs591058	Additive model	1.24	1.02–1.51	0.032	Increased risk
*IL1B*	rs16944	Recessive model	1.36	1.03–1.81	0.033	Increased risk
*CASP3*	rs4647693	Dominant model	4.76	1.51–15.07	0.008	Increased risk
*VDR*	rs2228570	Dominant model	1.52	1.10–2.11	0.012	Increased risk
*VDR*	rs2228570	Additive model	1.22	1.00–1.48	0.047	Increased risk
*ARNTL (BMAL1)*	rs7949336	Dominant model	1.49	1.06–2.09	0.021	Increased risk
*CCDC26*	rs6651255	Dominant model	0.51	0.29–0.91	0.021	Reduced risk
*CCDC26*	rs6651255	Additive model	0.76	0.59–0.98	0.034	Reduced risk
*CCDC26*	rs7816342	Dominant model	0.48	0.28–0.84	0.009	Reduced risk
*CEP162/RIPPLY2*	rs62420748	Recessive model	0.58	0.35–0.96	0.034	Reduced risk
*CEP162/RIPPLY2*	rs62420748	Additive model	0.77	0.61–0.96	0.024	Reduced risk
*LRP5*	rs3781579	Dominant model	0.62	0.40–0.96	0.032	Reduced risk
*LRP5*	rs3781579	Additive model	0.64	0.42–0.98	0.039	Reduced risk
*GDF5*	rs143383	AA vs. GG	0.56	0.33–0.95	0.032	Reduced risk
*GDF5*	rs143383	Additive model	0.76	0.61–0.96	0.018	Reduced risk

Note: Odds ratios, 95% confidence intervals, and *p*-values were calculated using multivariable logistic regression models under the indicated genetic models. Models were adjusted for age, BMI, education level, smoking exposure, family history of disc-related disease, weight-bearing workload, and duration of bending work. Only SNPs with nominal associations are shown in this table; full association results are provided in Supplementary Table S1. IDD, intervertebral disc degeneration; SNP, single-nucleotide polymorphism; OR, odds ratio; CI, confidence interval. Because multiple candidate SNPs and genetic models were evaluated, these associations should be interpreted as nominal and exploratory.

**Table 3:** Linkage disequilibrium and haplotype association analysis.

Analysis	Gene/Region	SNP Pair/Block	Haplotype	Frequency	Estimate	95% CI	*p*-Value
LD analysis	*ACAN*	rs1042630–rs3825994	—	—	r^2^ = 0.857	—	—
LD analysis	*CHST3*	rs1871452–rs4148946	—	—	r^2^ = 0.959	—	—
LD analysis	*CCDC26*	rs6651255–rs7816342	—	—	r^2^ = 0.939	—	—
Haplotype analysis	*CCDC26*	rs6651255–rs7816342	AC	0.730	0.87	0.76–0.99	0.046

Note: Linkage disequilibrium was evaluated using r^2^. Haplotype associations with IDD risk were estimated using logistic regression models. Only SNP pairs with strong linkage disequilibrium and the nominally significant haplotype are shown in this table; full linkage disequilibrium and haplotype results are provided in Supplementary Table S3. LD, linkage disequilibrium; IDD, intervertebral disc degeneration; OR, odds ratio; CI, confidence interval.

**Table 4:** Key gene–gene interaction results.

SNP Combination	Interaction Scale	Main Indicator	Estimate	95% CI	*p*-Value	Interpretation
*ACAN* rs698621 × *CASP3* rs4647693	Additive	RERI	1.29	0.22–2.36	—	Synergistic
*ACAN* rs698621 × *VDR* rs2228570	Additive	RERI	0.74	0.26–1.22	—	Synergistic
*ACAN* rs698621 × *CEP162/RIPPLY2* rs62420748	Additive	RERI	0.49	0.08–0.91	—	Synergistic
*IL1B* rs16944 × *VDR* rs2228570	Additive	RERI	0.90	0.69–1.11	—	Synergistic
*CASP3* rs4647693 × *CEP162/RIPPLY2* rs62420748	Additive	RERI	0.85	0.56–1.14	—	Synergistic
*ACAN* rs698621 × *CEP162/RIPPLY2* rs62420748	Multiplicative	Interaction OR	2.08	1.07–4.02	0.030	Synergistic
*IL1B* rs16944 × *VDR* rs2228570	Multiplicative	Interaction OR	5.55	2.34–13.15	<0.001	Synergistic
*VDR* rs2228570 × *GDF5* rs143383	Multiplicative	Interaction OR	3.96	1.05–14.93	0.042	Synergistic
*MMP3* rs591058 × *CEP162/RIPPLY2* rs62420748	Multiplicative	Interaction OR	0.18	0.07–0.46	<0.001	Antagonistic
*CASP3* rs4647693 × *VDR* rs2228570	Multiplicative	Interaction OR	0.05	0.0001–0.55	0.015	Antagonistic
*LRP5* rs3781579 × *CEP162/RIPPLY2* rs62420748	Multiplicative	Interaction OR	0.35	0.15–0.84	0.019	Antagonistic

Note: Additive interaction was assessed using the relative excess risk due to interaction. Multiplicative interaction was assessed using logistic regression models with product terms. Models were adjusted for age, BMI, education level, smoking exposure, family history of disc-related disease, weight-bearing workload, and duration of bending work. Interaction estimates with wide confidence intervals should be interpreted cautiously. Detailed gene–gene interaction results are provided in Supplementary Table S4. RERI, relative excess risk due to interaction; OR, odds ratio; CI, confidence interval.

**Table 5:** Key gene–environment interaction results.

SNP–Environment Combination	Interaction Scale	Main Indicator	Estimate	95% CI	*p*-Value	Interpretation
*MMP3* rs591058 × smoking	Additive	RERI	0.85	0.15–1.56	—	Synergistic
*IL1B* rs16944 × duration of bending work	Additive	RERI	1.33	0.04–2.61	—	Synergistic
*CASP3* rs4647693 × smoking	Additive	RERI	2.46	0.37–4.55	—	Synergistic
*ARNTL* (*BMAL1*) rs7949336 × duration of bending work	Additive	RERI	1.42	0.14–2.70	—	Synergistic
*MMP3* rs591058 × smoking	Multiplicative	Interaction OR	1.94	1.01–3.73	0.045	Synergistic
*CASP3* rs4647693 × weight-bearing workload	Multiplicative	Interaction OR	9.51	1.90–47.53	0.006	Synergistic
*LRP5* rs3781579 × weight-bearing workload	Multiplicative	Interaction OR	0.38	0.16–0.90	0.027	Antagonistic
*GDF5* rs143383 × smoking	Multiplicative	Interaction OR	0.37	0.17–0.81	0.013	Antagonistic

Note: Additive interaction was assessed using the relative excess risk due to interaction. Multiplicative interaction was assessed using logistic regression models with product terms. Gene–environment interaction models were adjusted for age, BMI, and education level. For additive interaction, statistical evidence was assessed using the 95% CI of RERI; therefore, *p*-values were not reported for RERI estimates. Detailed results for the selected gene–environment interaction signals are provided in Supplementary Table S5. RERI, relative excess risk due to interaction; OR, odds ratio; CI, confidence interval.

## Data Availability

The data presented in this study are available from the corresponding author upon reasonable request.

## Supplementary Materials

The following supporting information can be downloaded at: https://www.mdpi.com/article/10.3390/genes17080967/s1, Supplementary Table S1. Full association results of retained candidate SNPs under different genetic models. Supplementary Table S2. Genotyping quality control, Hardy–Weinberg equilibrium, and inclusion status of the 60 initially selected candidate SNPs. Supplementary Table S3. Linkage disequilibrium and haplotype analyses. Supplementary Table S4. Detailed gene–gene interaction results among selected candidate variants. Supplementary Table S5. Selected gene–environment interaction signals. Supplementary Table S6. GMDR model performance and selected high-order interaction models. Supplementary Table S7. Selection rationale and prior evidence for the 60 initially selected candidate SNPs.
